# Maximising Efficient Endoscopy Training—Implementation Analysis of a Tool to Improve Endoscopy Training

**DOI:** 10.1055/a-2895-3166

**Published:** 2026-07-03

**Authors:** Catherine Eley, Neil D. Hawkes, Richard John Egan, Wyn Griffith Lewis

**Affiliations:** 1 General Surgery 659853 The Grange University HospitalCwmbranWalesUnited Kingdom of Great Britain and Northern Ireland; 2 518564 NHS Wales Health Education and Improvement WalesNantgarwWalesUnited Kingdom of Great Britain and Northern Ireland; 3 97692 Royal Glamorgan HospitalLlantrisantWalesUnited Kingdom of Great Britain and Northern Ireland; 4 97701 Morriston HospitalSwanseaWalesUnited Kingdom of Great Britain and Northern Ireland; 5 7759 Swansea UniversitySwanseaWalesUnited Kingdom of Great Britain and Northern Ireland; 6 North Wales Medical School, Bangor UniversityBangorWalesUnited Kingdom of Great Britain and Northern Ireland; 7 97609 University Hospital of WalesCardiffWalesUnited Kingdom of Great Britain and Northern Ireland; 8 School of Surgery 518564 NHS Wales Health Education and Improvement WalesNantgarwWalesUnited Kingdom of Great Britain and Northern Ireland

**Keywords:** quality and logistical aspects, training, quality management

## Abstract

**Introduction** UK demand for gastrointestinal (GI) endoscopy exceeds capacity, with trainee access to dedicated training lists below the Joint Advisory Group on GI Endoscopy (JAG) recommendation of ≥20 lists per year [1–5]. Barriers include rota conflicts and limited trainer availability, which restrict equitable access [6–12]. Improving visibility of training opportunities may improve access to supervised procedures.

**Methods** The Maximising Efficient Endoscopy Training (MEET) tool is a web-based planner built using Laravel PHP and the TALL stack. It matches trainees to suitable training lists and records reasons for missed sessions. Piloted in a district general hospital in Wales, feasibility was examined using the ERIC framework, Programme Theory, and PRISM. A six-week retrospective review of endoscopy activity was compared with a six-week simulated prospective phase; results are displayed as counts, percentages, and 95% confidence intervals. Narrative and Likert feedback captured user perspectives.

**Results** Potential dedicated training lists rose two-fold (19.2–41.7%) and potential ad hoc training opportunities four-fold (14.3–66.7%). Residents engaged readily; trainers required targeted onboarding. Barriers included perceived administrative burden, digital literacy variation, and misalignment between consultant and trainee schedules. Managers valued MEET’s ability to highlight available backfilling.

**Conclusion** MEET may offer a scalable, adaptable approach to identifying training opportunities. Although the findings arose from a simulated environment, the exercise provided insights into implementation barriers that can inform and strengthen subsequent real-world deployment. Further evaluation in routine clinical settings is necessary to determine its potential to support progression towards JAG certification and Certificate of Completion of Training (CCT).

## Introduction

Demand for gastrointestinal (GI) endoscopy in the United Kingdom continues to exceed capacity, driven by an ageing population, expanded surveillance programmes, and earlier disease-detection initiatives. 1 2 Rising waiting times highlight the need to expand workforce capacity across the full spectrum of diagnostic and therapeutic endoscopy. 3 A recent UK survey of the endoscopy workforce highlighted an urgent training need to address expected shortfalls due to retirement and called for a workforce plan to support training efficiency and optimise endoscopist job planning, with the aim of meeting service needs and developing a sustainable workforce. 4 Better training may also enhance endoscopist performance and patient safety. 5

The Joint Advisory Group on GI Endoscopy (JAG) certifies all UK endoscopists and recommends each trainee receive at least 20 dedicated training lists annually. 6 In 2019, hospitals in Wales fell 35% short of this standard, with a median of 13 lists per trainee. 7 Although learning curves for upper GI endoscopy and colonoscopy are well established, 8 9 10 only half of the trainees access the recommended training allocation. 11 Barriers include conflicting clinical commitments, lack of dedicated trainer allocation, competition for training lists, and inter-specialty disparities. 12 Limited access delay progression reduces JAG certification rates—especially in colonoscopy—and restricts opportunities for advanced procedures such as ERCP. Shortened specialty training under Shape of Training reforms has exacerbated these issues. 13

These limitations threaten workforce sustainability, particularly with high consultant retirement rates predicted in the next five years and projected gaps in screening colonoscopy and specialist services. 11 12 13 14 15 The number and quality of training lists is therefore a key indicator of training health within an endoscopy unit. Yet, analysis of the National Endoscopy Database shows only 6% of 1.64 million procedures are allocated to training, 3 likely worsened by an increased reliance on out-of-hours and private sector lists to address the waiting list backlog. Trainers have called for better data on reasons for trainee non-attendance. 16 Planning tools could optimise use of training list time by reallocating an alternative trainer or trainee if either are absent—or identify where a training list cannot proceed, so that list space can be backfilled to maintain service capacity. An effective system should allow forward planning across multiple contexts, record reasons for missed training, support real-time stakeholder input, and highlight reasons for professional resistance and IT-related barriers to adoption.

## Methods

### Design

The Maximising Efficient Endoscopy Training (MEET) Tool is a web-based list planning system. Stakeholders included gastroenterologists, surgeons, nurse endoscopists, trainees, unit administrators, and managers.

Built with the Laravel PHP framework and TALL stack (Tailwind CSS, Alpine, Laravel, Livewire) and hosted on Microsoft Azure, MEET matches trainees to available training lists by common training domain (e.g. diagnostic colonoscopy, upper GI endoscopy). Six user profiles were defined according to JAG terminology—Planner, Training Lead, Trainer, Trainee, Viewer, and Administrator.

**Fig. 1:**
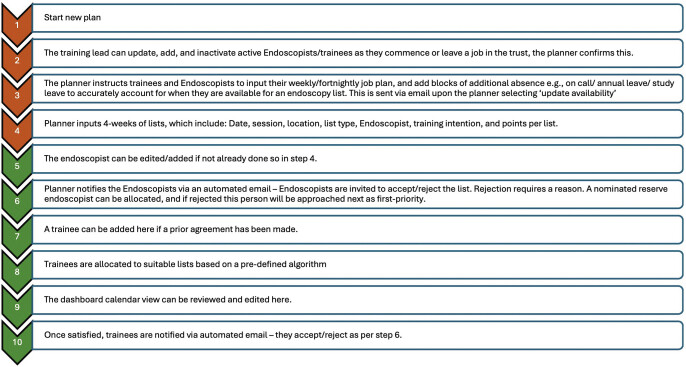
10-step planning process—MEET Tool.

The Planner completes a ten-step process in two stages ( Fig. 1 ; **Supplementary Fig. 1** ). The development phase highlighted the need to input schedules and job plans to support accurate matching and identify staff to backfill unallocated lists. Trainers and trainees can accept or decline lists, selecting a predefined reason for non-attendance so as to inform service and training planning.

### Pilot Site

The tool was piloted in a JAG accredited district general hospital in Wales with a two-room endoscopy suite and 14 endoscopists (six surgeons, six gastroenterologists, two clinical endoscopists). The aim was to evaluate functionality, gather user feedback, and explore potential impact on training and service provision.

## Outcomes

**Primary Outcome**

- Change in the number and proportion of potential dedicated training lists utilised before and after MEET implementation.

**Secondary Outcomes**

- Change in the number and proportion of potential ad-hoc training opportunities
- User acceptability and perceived feasibility (Likert-scale and narrative feedback)
- Feasibility of tool operation within a simulated environment.

### Implementation Feasibility Exercise

**Fig. 2:**
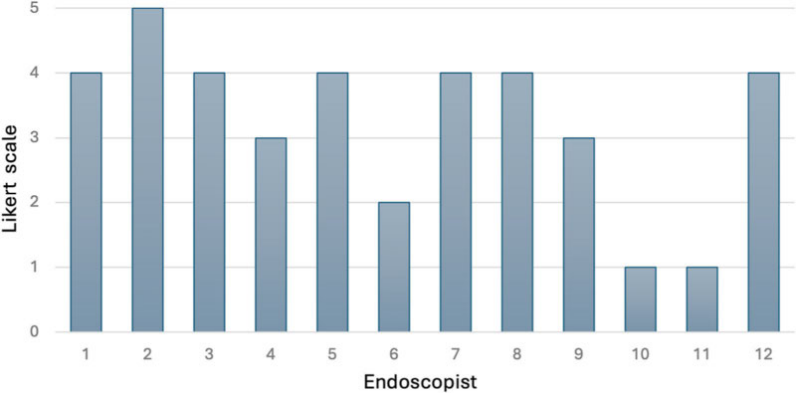
Endoscopists initial attitude to MEET implementation (Likert scale 1–5: one—unwilling to engage, five—very keen to engage).

A user manual was provided and in-person sessions offered to all. Residents attended training and successfully entered data; trainers required more individualised support due to scheduling constraints. Narrative and Likert-scale feedback (1 = very reluctant, 5 = very keen) was collected ( Fig. 2 ).

Implementation was analysed using the Expert Recommendations for Implementing Change (ERIC) framework, Programme Theory, and the Practical Robust Implementation and Sustainability Model (PRISM), focusing on stakeholder engagement, adaptability, behavioural influences, and feedback loops.

### Evaluation

A six-week retrospective review of endoscopy lists used the MEDILOGIK Endoscopy Management System (EMS) and endoscopy reports to record list type (training or service) and trainer/trainee presence. Service lists involving trainees were classified as ad hoc training. Dedicated training lists were delivered by one of seven endoscopists accredited as JETS trainers; the remaining seven had all completed Train the Trainer courses with credentials to supervise trainees and act as trainers, ad-hoc, in the original trainer’s absence to preserve training opportunities.

This was followed by a six-week feasibility exercise with one user acting in all roles in a test environment, initiating the ten-step plan; responding to prompts; and entering job plans, on-call rotas, and leave. Data collected included list type and trainer/trainee potential presence based on recorded availability.

Results are displayed as counts, percentages, and 95% confidence intervals using SPSS v31 (IBM Corp, Armonk, NY).

## Results

The number of endoscopy lists was similar across the retrospective and prospective six-week periods. The primary outcome demonstrated a two-fold increase in potential dedicated training list capacity from five (19.2%, (3–35%)) to ten (41.7%, (20–63%)) using the tool. Identified potential ad hoc training opportunities increased four-fold, from 10 (14.3%, 5.9–22.7%) to 44 (66.7%, 55–78.3%) ( Table 1 ). These findings suggest that MEET may help identify additional training opportunities, although confirmation in a real-world setting is required. The tool functioned reliably within the test environment, accessible via all modern browsers and available as an app icon for iOS and Android.

**Table 1:** Endoscopy utilisation 6-weeks prior to and following feasibility implementation exercise.

	6-week period before		6-week period after	
		95% CI (%)		95% CI (%)
All lists	114		108	
Training lists	26		24	
Service lists	70		66	
Bowel Screening lists*	18		18	
Training list with trainee	5 (19.2%)	3–35	10 (41.7%)	20–63
Ad-hoc training opportunity	10 (14.3%)	5.9–22.7	44 (66.7%)	55.0–78.3

* Exempt from training.

### User Feedback

Residents viewed MEET positively and entered their data successfully. Trainers required more personalised, flexible support; none attended the group session. Likert-scale scores (1 = unwilling, 5 = very keen) are shown in Fig. 2 . Trainers generally recognised the tool’s potential benefits but some—particularly those not identifying as trainers—feared it would commit them to training.

The most frequent concern was the perceived burden of entering data into an additional system. Some suggested secretarial support for data entry; others preferred existing paper-based absence reporting. Directorate managers supported the concept, mainly for its potential to identify available staff to backfill sessions.

Across all roles, high workload was a barrier to adoption. While residents saw clear benefits and trainers acknowledged its potential, the need for initial time investment without an immediately visible return limited engagement.

## Discussion

This pilot suggests that MEET could help identify additional training opportunities with a two-fold increase in potential dedicated training lists and increasing potential ad hoc opportunities four-fold. Despite this, the operational paradox also identified latent training opportunities that, despite the tool, remained unfilled. Beyond scheduling, the tool captures quantitative data on reasons for missed training, turning anecdotal reports into actionable intelligence, which may support more targeted interventions. Such data could help identify specific barriers and inform local and regional leads to address specific barriers and support reorganisation of ad-hoc endoscopy lists into formal, JAG-compliant training lists.

**Fig. 3:**
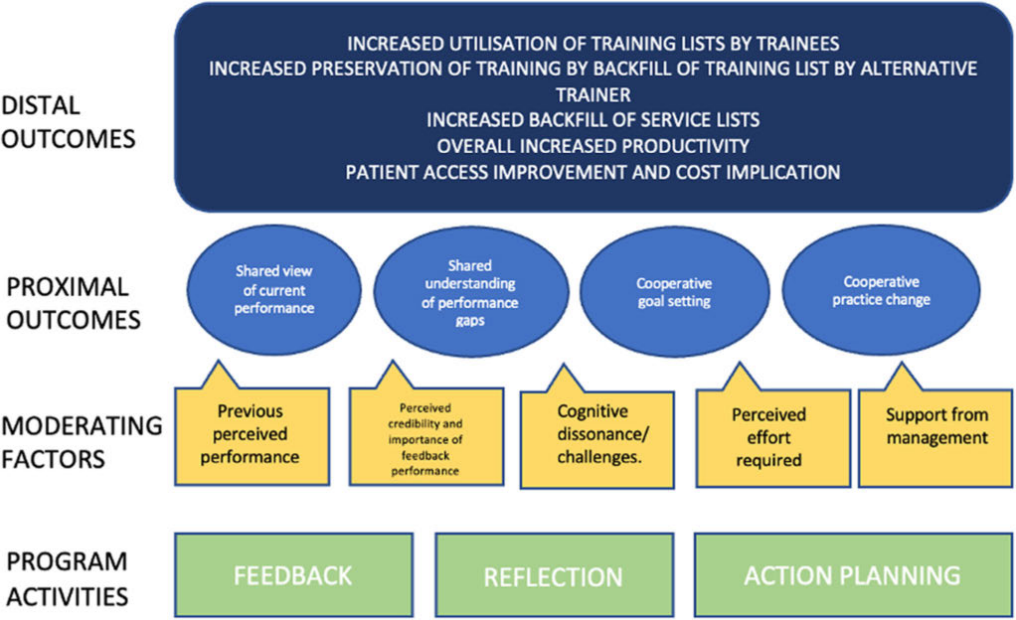
Programme theory of an intervention (MEET Tool) to improve productivity of the endoscopy department (adapted from ref. 17 ).

Real-world implementation challenges were consistent with those seen in healthcare innovation more broadly: technology adoption depends as much on human factors as technical functionality. The ERIC framework, while complex, offered insights into the active components of MEET tool implementation ( Table 2 , Appendix 1), and programme theory helped identify strategies that facilitated uptake, including stakeholder engagement, iterative development, and provision of user manuals and training sessions 17 ( Fig. 3 ).

**Table 2:** ERIC framework domains applicable to MEET tool.

Domain	Application to MEET tool
Conduct local needs assessment	Training capacity in Wales: underperforming when compared to JAG standards (7) 66% underutilisation of training lists in the pilot hospital
Assess for readiness and identify barriers and facilitators	Barriers: Resistance to change. Digital literacy required. Data input demand, when already under high work time-pressure Strengths: Generally accepted vision of the potential for improvement. Support from directorate management
Create a learning collaborative	Attempted addition of a second more digitally literate member of the administrative team aimed to inspire collaborative learning culture
Develop educational materials	Educational guide developed and adapted for each user group
Distribute educational materials	User guide sent to all users. Concept and development presented at surgical and gastroenterology educational conferences
Conduct educational meetings	Targeted stakeholder meetings with administrators, endoscopists, and trainee endoscopists, to provide demonstrations and opportunity for hands-on practice
Make training dynamic	Hands-on practice as above
Centralize technical assistance	Technical assistance provided ‘on the ground’ by CE with direct level of communication with digital team.
Local technical assistance	As above
Identify and prepare champions	Engaged individuals need identifying for scale-up
Conduct cyclical small tests of change	The tool was developed in a ‘test area’ with weekly feedback meetings and iterative tool development
Audit and provide feedback	Results need feeding back to users to inspire change and encourage behaviour modification
Purposely re-examine the implementation	Results and feedback inform adjustments to improve the intervention
Conduct local consensus discussions	Feedback was recorded during educational meetings and acted on accordingly during iterative development
Develop a formal implementation blueprint	Appendix 1
Remind clinicians	Fortnightly reminders sent by the planner through the tool to encourage engagement. Feedback was intended to be provided to all users to demonstrate the MEET tool’s role in improving access to training

Despite this, adoption was uneven. Residents engaged readily, while trainers needed more personalised onboarding. Qualitative feedback highlighted the interplay between workload pressures and the perceived burden of digital change. Although the tool was conceptually acceptable, its implementation collided with established, familiar low-effort practices that relied less upon the endoscopist and more on administrative staff. Mismatches in digital literacy and reluctance to change established paper-based processes further limited engagement. Some endoscopists were hesitant to participate due to concerns about increased training obligations. Where the tool identified endoscopists able to backfill and preserve training, with appropriately motivated and digitally literate support, it was possible to remove the trainee from the list, if backfilled by an endoscopist reluctant to train, protecting their perceived clinical competencies and risk profiles. The cumulative effect of these barriers resulted in reduced engagement, inconsistent data entry, and a widening gap between MEET’s ability to identify capacity and the organisations’ ability to act on it. The PRISM model analysis reinforces the importance of aligning technical capacity, organisational support, and user willingness 18 ( Table 3 ). Sustained use may require structured change management approaches such as the ADKAR framework, ensuring that awareness, desire, and ability are built into rollout strategies. 19

**Table 3:** PRISM analysis of the MEET tool. Adapted from Ref. 18 .

INPUTS		
Technical factors	Complexity of procedures	Planner: follows 10-step data input, list planning ( **Supplementary Fig. 1** ) Resident/Endoscopist: inputs job plans, availability and absences using regular automated reminders
	Health information system design	Integrated list planning, built with open-source PHP framework Laravel and TALL Stack, and cloud hosted with Microsoft Azure
	Computer software	Browser-based platform with an App icon available on iOS and Android
	IT complexity	Integrated with University Health Board (UHB) IT systems and can be used by UHB staff through existing hardware and software systems. Requires system password
Organisational factors:	Governance	Accepted by HEIW. As no patient data collected, information governance did not apply
	Planning	Iterative development, with regular collaborative feedback of stakeholders to reflect, learn, and improve on earlier iterations. Prioritize the steps with most potential, in a resource-limited environment, to reach and benefit the widest audience most efficiently 21
	Resources	Requires administrative time
	Training	Step-by-step guide plus hands-on/remote support
	Supervision	Hands-on and remote support: reflection on training needed and support user engagement
	Finances	Financially supported by HEIW, with digital team for initial development. Grant awarded by the National Endoscopy Project (NEP) to support further development and dissemination
	Information distribution	Demonstrate a shared view of current performance, perceived areas for improvement, intended distal outcomes. Feedback of progress
	Promotion of culture of information	Promoting understanding of the value of endoscopic training, current deficit in availability of training lists, and appreciation of how the MEET tool can maximise the benefits of timetabled training lists
Behavioural factors	Data demand	For data process to succeed, endoscopists and trainee endoscopists must accurately update their availability: annual leave, study leave, and other competing commitments
	Data quality checking skill	Requires regular interaction with the tool and fortnightly reminder emails to users to update their availability information
	Problem solving for HIS tasks	Direct line of communication with digital team
	Competence in HIS tasks:	Requires digital literacy
	Confidence levels for HIS tasks	Requires digital literacy and engagement to embrace change
	Motivation	Engagement required to embrace change
**PROCESSES**		
Processes	Data collection	Endoscopy availability input by endoscopists and trainee endoscopists Endoscopy list plan input by administrator
	Data transmission	Presented via live dashboard
	Data processing	Designed with an Administrator function, which allows for regular review of user inputs to the system and push notifications
	Data analysis	Design allows for analytics to be applied to the inputted training data, enabling percentages of utilised lists and reasons why residents and trainers could not attend lists
	Data display	Live dashboard view of endoscopy list plans Visibility of trainer and trainee availability Lists available for backfill identified
	Data quality checking	Inputs into the system are dictated by selection of pre-determined options standardising data inputs
	Feedback	Users can contact system administrators for active support. During the implementation pilot, users were also able to log questions with the study investigators and were given direct support where required
**OUTPUTS**		
Improved Performance	Data quality	Accurate data related to each planned list, with prospectively collected reasons for non-attendance
	Information use	Utilisation of training lists by trainees Preservation of training by backfill of training list by alternative trainer Backfill of service list in absence of resident Overall productivity
**OUTCOMES**		
Increased utilisation of training list by trainees Increased preservation of training by backfill of training list by alternative trainer		
**IMPACT**		
Increased training delivery Overall increased productivity		

The study has several inherent limitations. It was conducted in a single NHS district general hospital, limiting generalisability to other settings or healthcare systems. The sample size was small and the observation period relatively short. Practical barriers prevented full real-time implementation and as a result data entry was completed by a single user and the prospective phase adapted within a simulated environment; results therefore reflect potential rather than actual changes in training delivery and so should be interpreted as exploratory; further evaluation in routine clinical settings is needed. Although this limits the ability to see real-time behavioural responses, the simulated exercise generated structured insights into the burden of data-entry and points of resistance. Nevertheless, the findings are useful to inform organisational change during the next implementation phase by showing where targeted support and administrative input may improve adoption and integration into routine practice.

Institutional policies also influenced implementation. NHS requirements for six weeks’ notice suited consultant schedules but were harder to match to residents’ more dynamic placements. While unavoidable last-minute changes are a reality, MEET’s prospective recording of such disruptions offers valuable evidence for service planning.

Despite these challenges, the tool’s design—simple, web-based, and adaptable—suggests potential for integration into regional training models now being implemented across the United Kingdom. The development of a user guide, identification of key stakeholders, and a clear implementation strategy may support wider adoption. Although demonstrated in a single institution, the technology could be applied across partner organisations or regional training networks, including procedural sub-speciality training, and may help identify gaps where logbook deficits hinder training progression. 20 Further scalability, such as linking MEET’s rota data with JETS, may streamline data entry and support equitable training evaluation.

## Conclusion

In settings where dedicated endoscopy training capacity is limited, MEET may offer a practical, adaptable approach to identify potential training opportunities. In this pilot, it suggested a possible increase in both dedicated and ad hoc opportunities, although these findings require confirmation in real-world practice. By capturing structured data on missed training, it may support evidence-based interventions rather than relying on anecdote.

Successful implementation depends on early stakeholder engagement, targeted trainer support, and strategies to overcome administrative resistance. Change management frameworks can help address these human factors, ensuring sustained use. With integration into regional training models, MEET may contribute to supporting training progression and workforce sustainability in endoscopy services, while also supporting progression towards achieving Certificate of Completion of Training (CCT).

## Supplementary Material

Ergänzendes Material Supplementary Material
