# Supplementary material for: Maximising Efficient Endoscopy Training—Implementation Analysis of a Tool to Improve Endoscopy Training
Source: Endosc Int Open. 2026 Jul 3;14:a28953166. doi: 10.1055/a-2895-3166 (PMC13334201; doi:10.1055/a-2895-3166)

Supplementary material: Maximising Efficient Endoscopy Training - implementation analysis of a tool to improve endoscopy training.

Appendix 1: Implementation blueprint.

Aim/purpose of the intervention:<br><br>Increase utilisation of training list by trainees<br><br>Increase preservation of training by backfill of training list by alternative trainer<br><br>Increase back-fill of service lists<br><br>Scope of the change<br><br>Increase training delivery<br><br>Increase overall productivity<br><br>Time frame and milestones<br><br>January 2022: initial IT development of the tool with weekly progress meetings until minimal viable product available for testing.<br><br>September 2022: test area tested → several suggestions for improvement acted on.<br><br>January 2023: Go ‘Live’ at the pilot site.<br><br>April/ May 2023 → barriers with lack of engagement from many users, required introduction of new member of the administrative team, with directorate support. Will needing training. Aim to help support existing team and mandate change<br><br>Appropriate performance/progress measures<br><br>Monthly review of MEET tool output and ‘real-world’ data.

Endosc Int Open | DOI 10.1055/a-2895-3166 | © The Author(s).

Supplementary Figure 1a: add new member

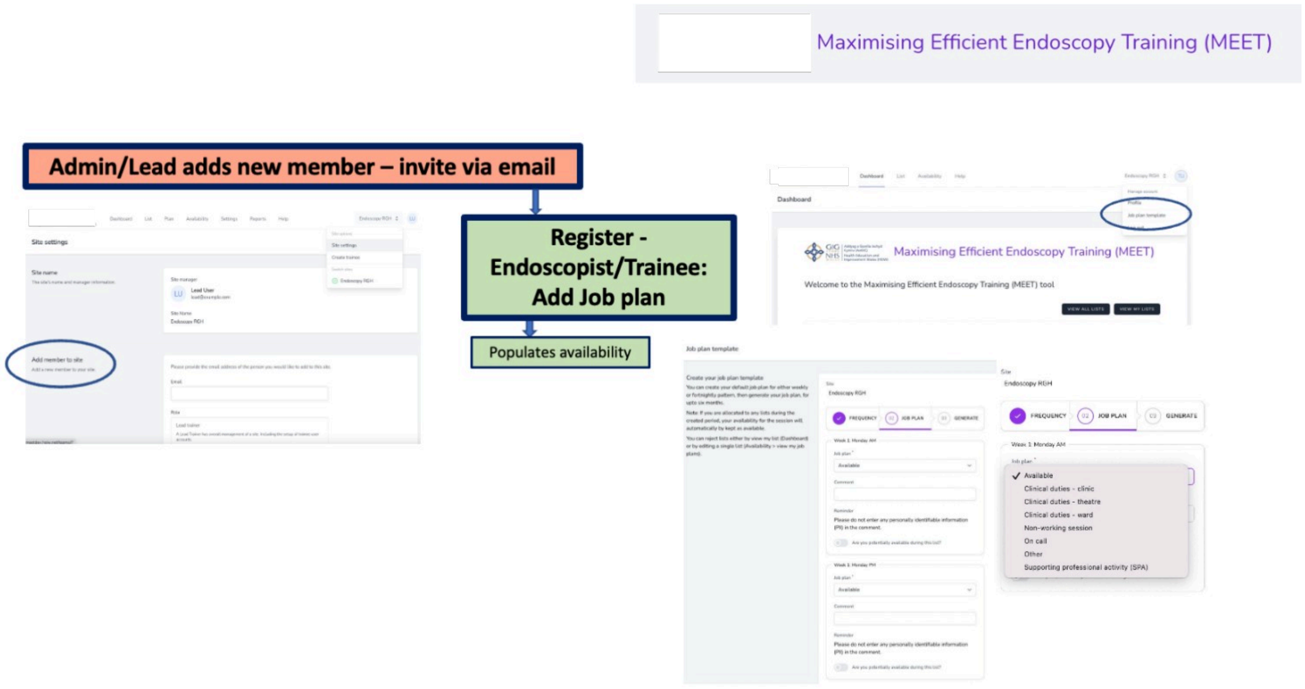

Supplementary Figure 1b: generate job plan

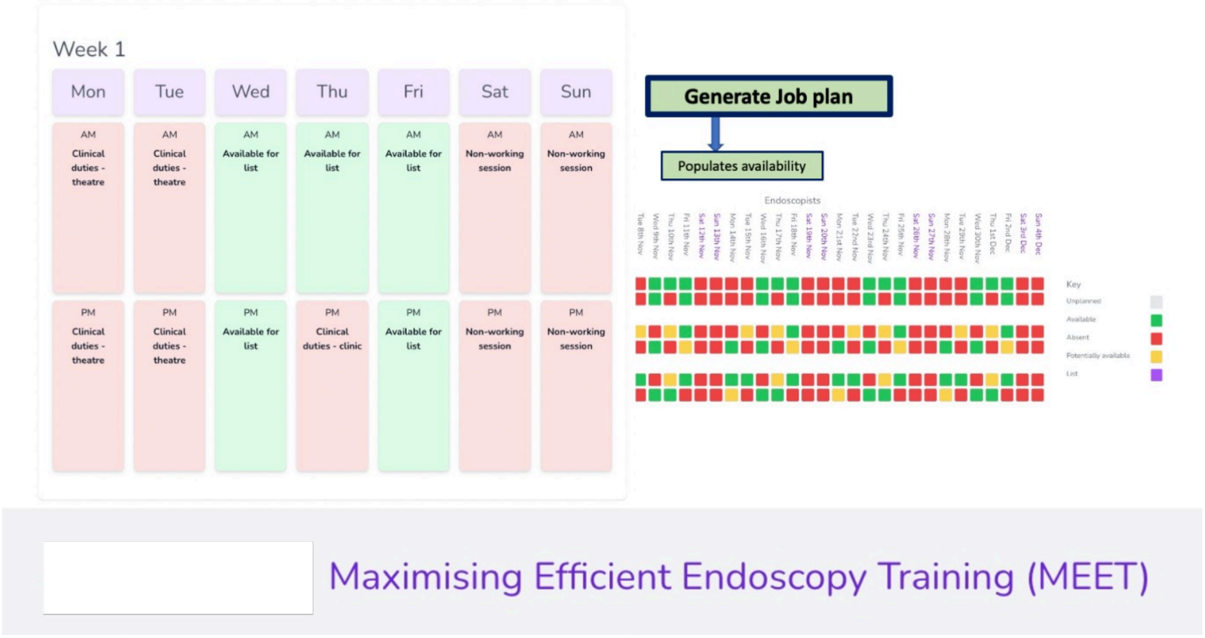

Supplementary Figure 1c: complete profile

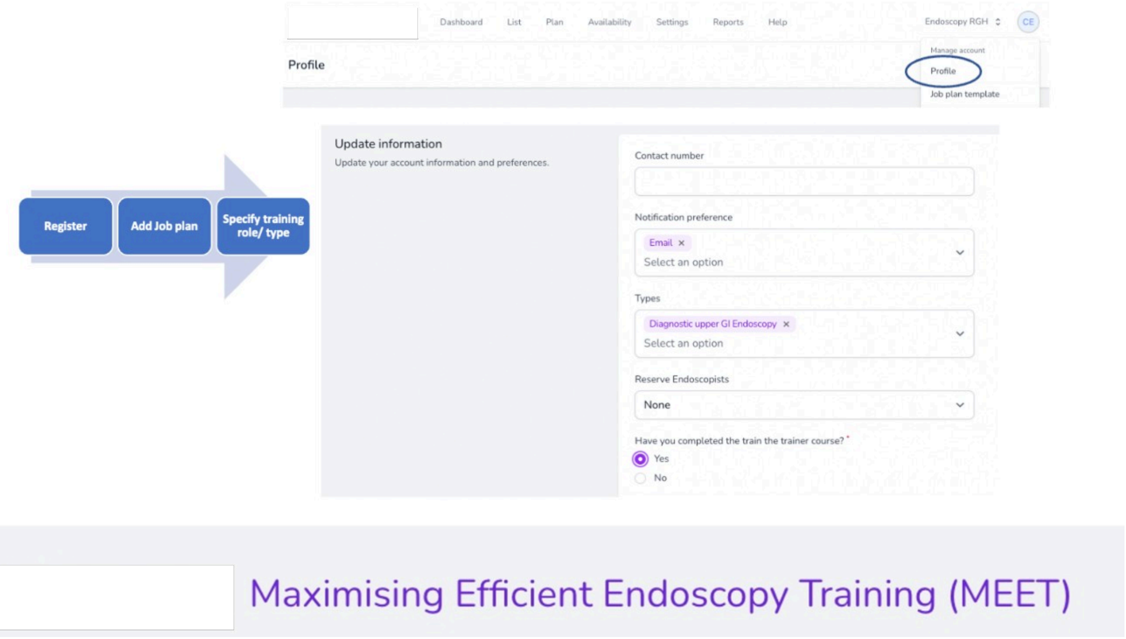

Supplementary Figure 1d: Step 1 - Start new plan

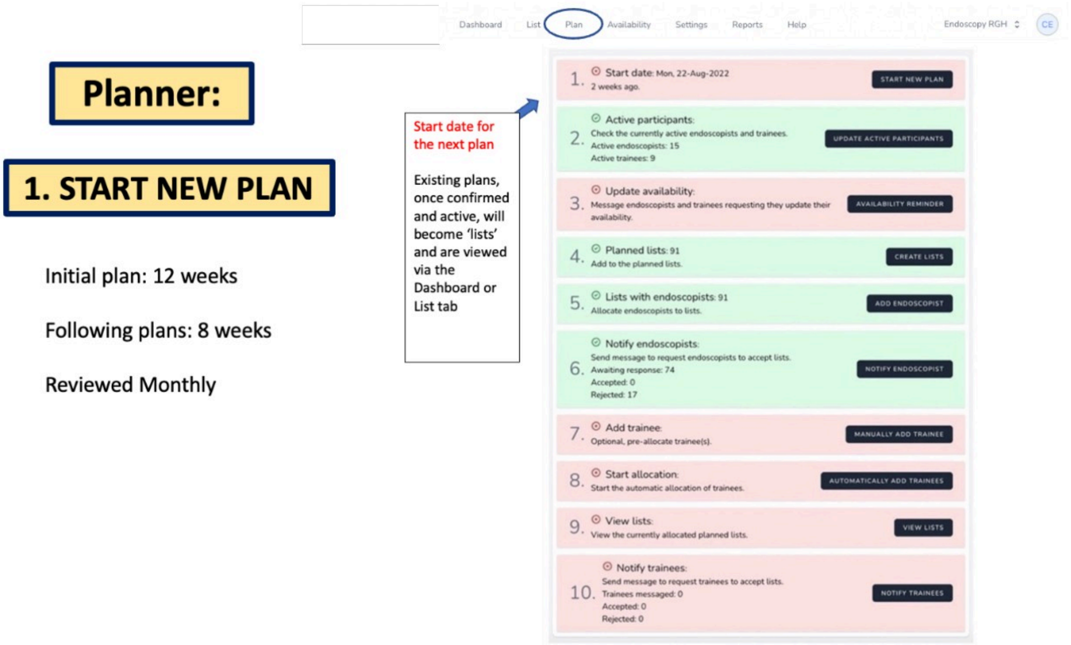

Supplementary Figure 1e: Step 2 - Review participants

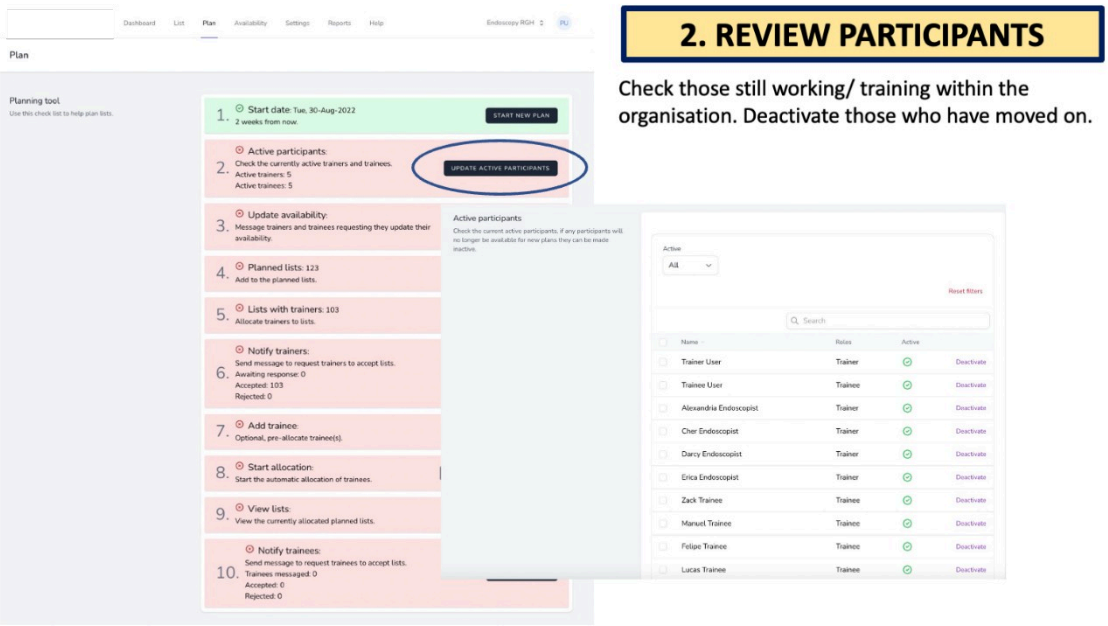

Supplementary Figure 1f: Step 3 - Update availability

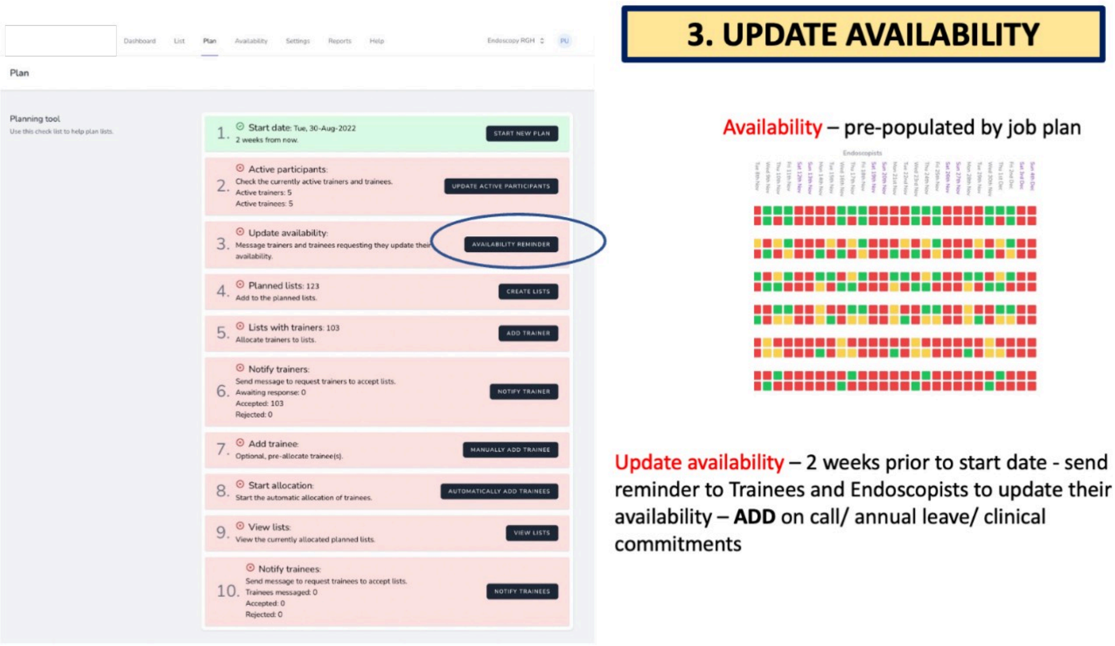

Supplementary Figure 1g: Step 3.1 - Update availability

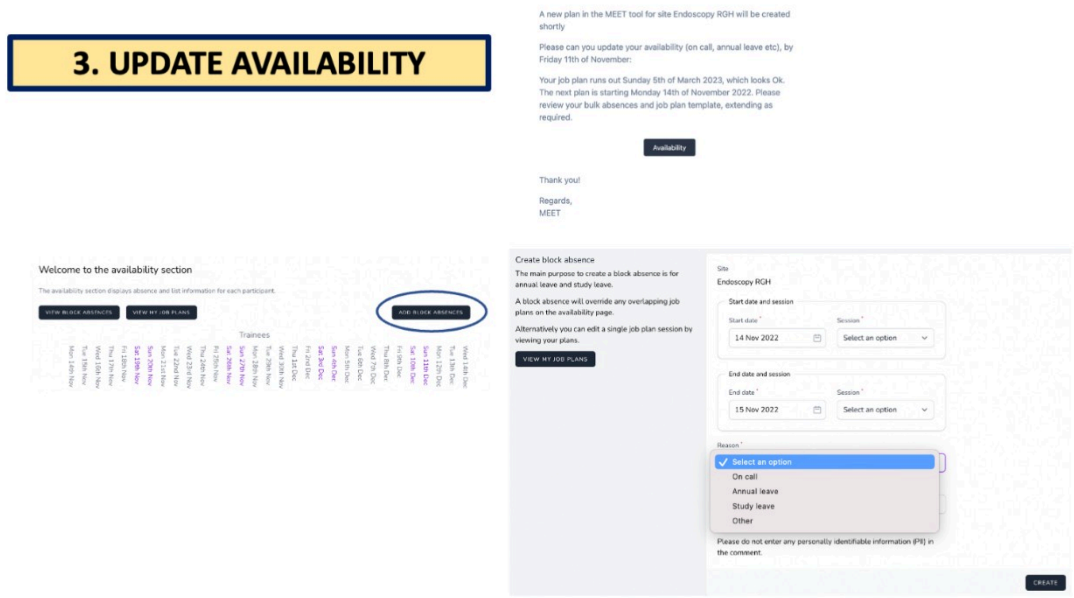

Supplementary Figure 1h: Step 4 - Create list

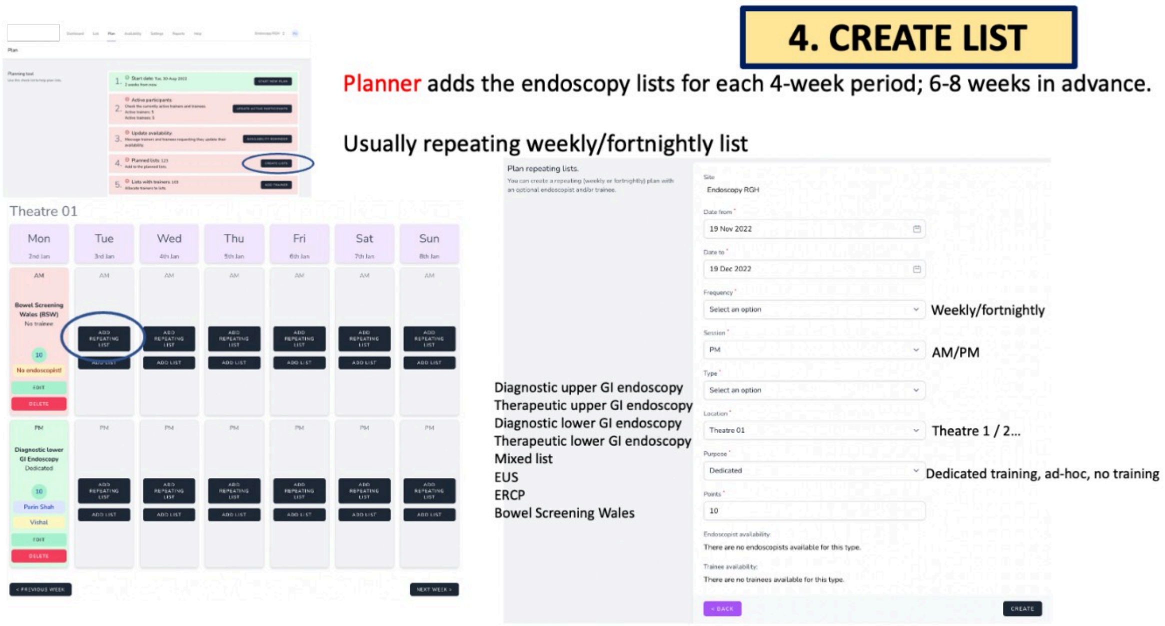

Supplementary Figure 1i: Step 5 - Add Endoscopist

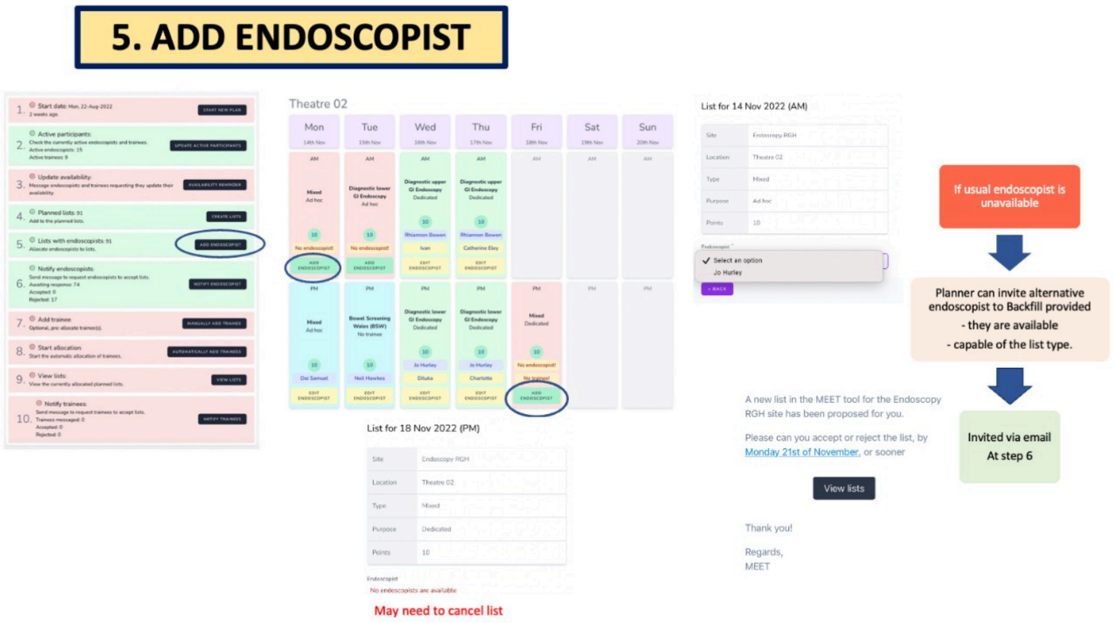

Supplementary Figure 1j: Step 6 - Notify Endoscopist

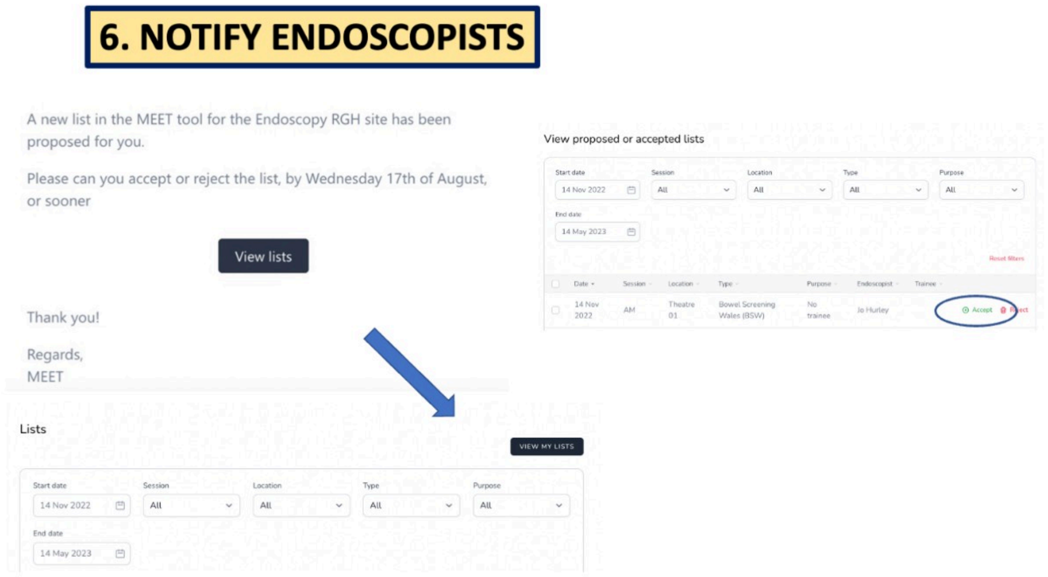

Supplementary Figure 1k: Step 7 - Add trainee

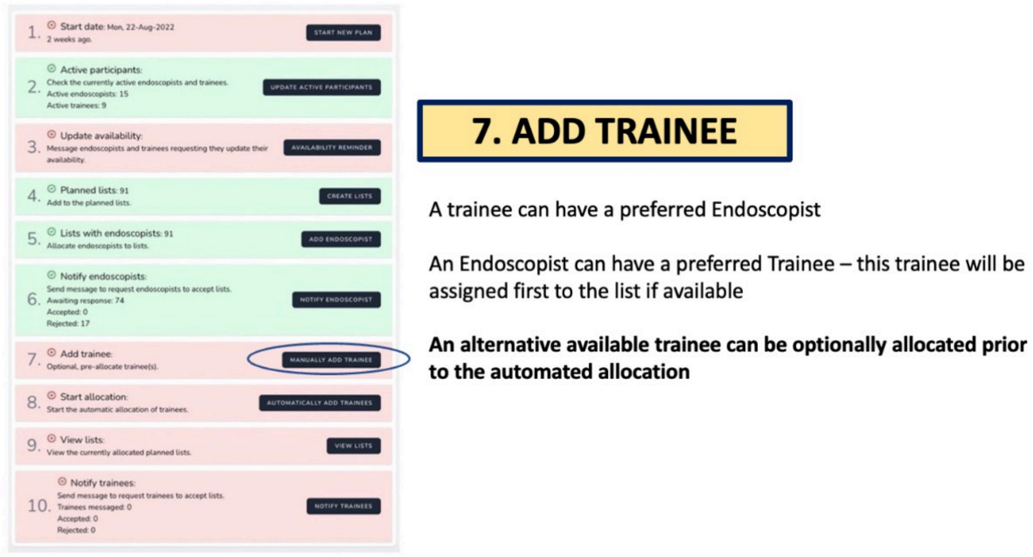

Supplementary Figure 1l: Step 8 - Start allocation

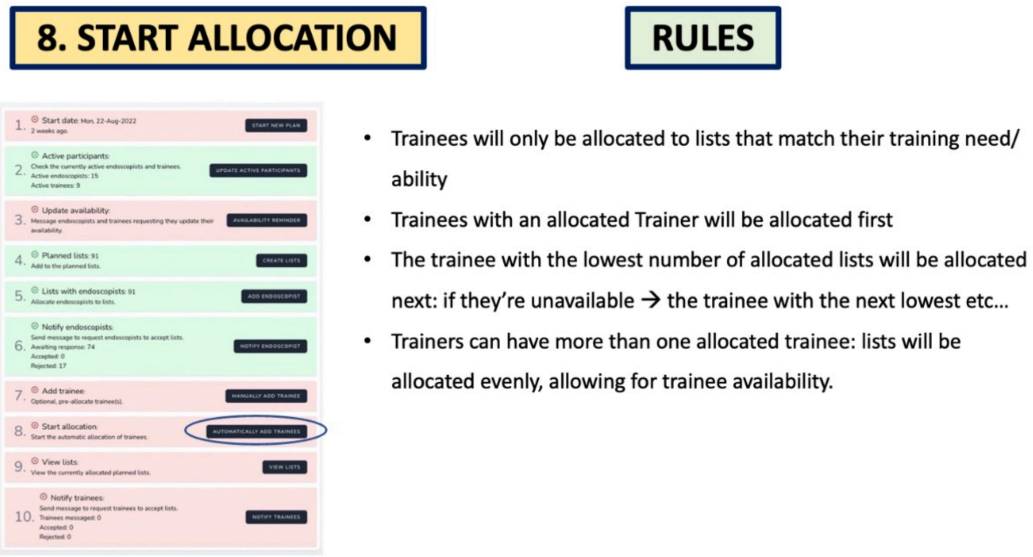

Supplementary Figure 1m: Step 9 - View lists

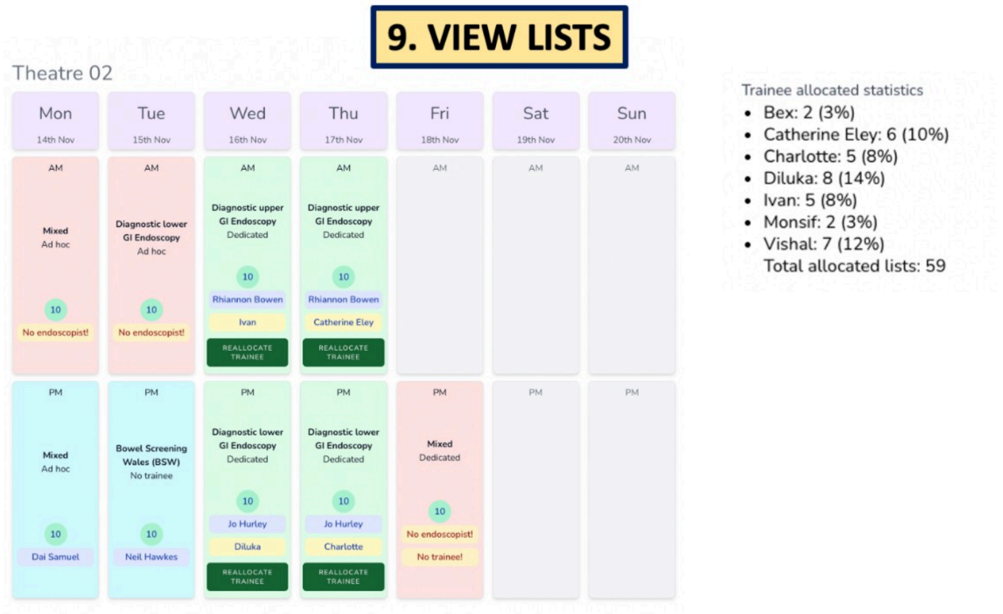

Supplementary Figure 1n: Step 10 - Notify trainees

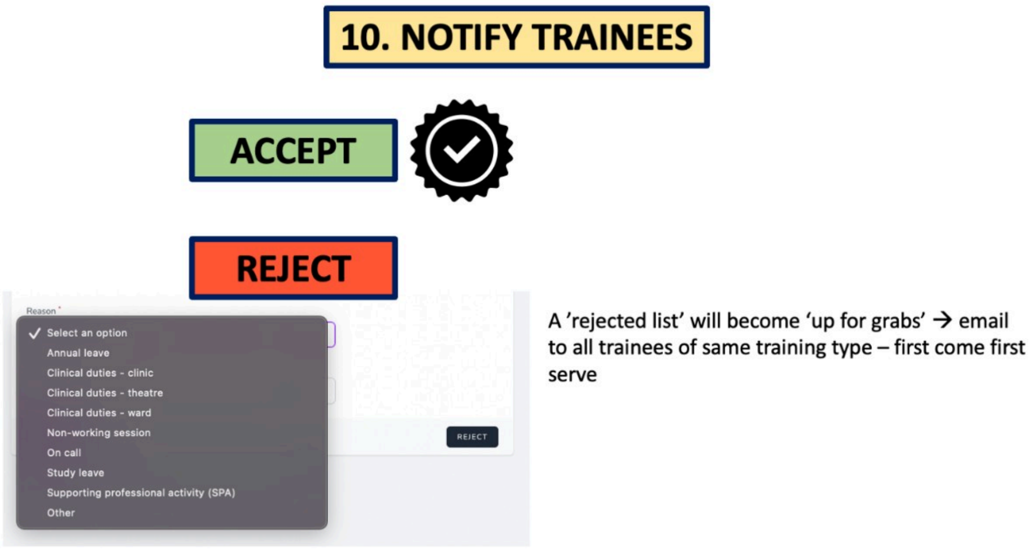

Supplement: Supplementary file 1 — Ergänzendes Material [file 10-1055-a-2895-3166_29011489.pdf]
